# Ensuring an inclusive global health agenda for transgender people

**DOI:** 10.2471/BLT.16.183913

**Published:** 2017-02-01

**Authors:** Rebekah Thomas, Frank Pega, Rajat Khosla, Annette Verster, Tommy Hana, Lale Say

**Affiliations:** a Gender, Equity and Human Rights Team, World Health Organization, avenue Appia 20, 1211 Geneva 27, Switzerland.; b Department of Public Health, Environmental and Social Determinants of Health, World Health Organization, Geneva, Switzerland.; c Reproductive Health and Research Department, World Health Organization, Geneva, Switzerland.; d HIV/AIDS Department, World Health Organization, Geneva, Switzerland.

There is a growing commitment in public health to understand and improve the health and well-being of transgender people and other gender minorities, who comprise an estimated 0.3–0.5% (25 million) of the global population.^1^ The adoption of *The* *2030 agenda for sustainable development* and its pledge to “leave no one behind” has given renewed impetus to this movement.^2^

Transgender is an umbrella term used to describe people with a wide range of gender identities, which are different from the sex assigned at birth. The term is increasing in familiarity globally, although other culturally-specific terms may be used to describe people who have non-gender binary identities, such as *hijra* (India), *waria* (Indonesia), *muxé* (Mexico), *fa’afafine* (Samoa), *kathoey* (Thailand) and Two-Spirit (indigenous North Americans). Many cultures and countries – including Australia, Bangladesh, Germany, India, Ireland, Nepal and Pakistan – recognize a third gender both in laws and in cultural traditions.

Transgender people share many of the same health needs as the general population, but may have other specialist health-care needs, such as gender-affirming hormone therapy and surgery. However, evidence suggests that transgender people often experience a disproportionately high burden of disease, including in the domains of mental, sexual and reproductive health. Exposure to violence, victimization, stigma and discrimination are also higher in this population. In addition, they experience barriers to accessing health care and health-determining resources, such as education, employment and housing.^3^ These barriers are largely attributable to legal, economic and social deprivation, marginalization, stigmatization and discrimination, including non-recognition of a gender identity that is different from the sex assigned at birth.

Recent debates have highlighted three challenges to the health and well-being of transgender populations. First, there are gaps in documenting evidence on the determinants and status of transgender people’s health. Second, transgender-specific health care and preferences must be better understood and barriers to access, including social and legal drivers of ill-health, tackled. Third, the underlying social exclusion mechanisms that undermine the right to health in health settings and broader society must be addressed.

Although the political debate on transgender people continues to be highly polarized, three major shifts are underway at the World Health Organization (WHO) that should contribute to tackling these challenges. These shifts are the proposed changes to relevant sections of the 11th edition of the *International statistical classification of diseases and related health problems (ICD-11)*; the adoption of a person-centred approach to transgender people’s health; and a shift towards an equity- and rights-based approach to the health of transgender people.

The first shift is the proposed revision of the ICD. Countries use the ICD to define eligibility and access to health services and as a basis for conceptualizing health conditions, treatments and outcomes. Health officials also use the ICD to facilitate the collection of data that guides policy and programme decisions. Under current proposals to the ICD-11 working group, transgender identities would no longer be classified as “Transsexualism” under the category of “Mental health and disorders” but would be classified as “Gender incongruence of adolescence and adulthood” under the category of “Conditions related to sexual health”. The proposed reclassification is expected to reduce the perception of illness and stigmatization of transgender people, and to lead the way for improvements in such course that transgender health can be understood, measured and addressed. The reclassification is also likely to positively affect how gender identity is viewed by society more broadly.^4^

Concerns have been expressed about the focus on sexual health in the new proposal and there have been calls for the total removal of gender identity from the ICD-11. However, the working group on the classification of sexual disorders and sexual health, which developed the proposal, acknowledges that inclusion in the ICD ensures transgender people’s access to gender-affirming health care as well as adequate health insurance coverage for such services. Recognition in the ICD also acknowledges the links between gender identity, sexual behaviour, exposure to violence and sexually transmitted infections. Questions remain among human rights advocates and some in the transgender community about the current proposal. However, the inclusion of transgender communities in the discussions of the proposal in various fora and field trials have provided a space for them to voice their concerns and contribute to the revision process.

The second shift marks a move from a disease-centred to a person-centred approach that puts the rights, preferences and voices of transgender people at the heart of policy and programming. Transgender health research has often focused on sexual health and especially on the human immunodeficiency virus (HIV). However, efforts to better understand the values and preferences of transgender populations reveal that many transgender people view discrimination in health care and access to quality, transgender-friendly and appropriate health-care services as more urgent health priorities.^5^ In response, WHO’s *Consolidated guidelines on HIV prevention, diagnosis, treatment and care for key populations* provides guidance on addressing the specific sexual health needs of transgender people and on identifying the social or environmental factors that affect access to quality and appropriate health services. The guidelines recognize both the intersections, but also the distinct differences between transgender people, men who have sex with men and sex workers. They set out essential strategies to work towards a more enabling environment, including the adoption of protective laws and policies, the decriminalization of consensual same-sex behaviour and legal recognition of transgender identities. They also outline best practices in the public provision of gender-affirming health care; training of health-care workers on respecting the human rights of transgender persons including the rights to dignity, privacy, autonomy and physical and psychological integrity; and prevention of gender-based violence.^6^

WHO and partners have developed a range of tailored guidance for health practitioners and policy-makers to better protect the health and rights of transgender people, including policy briefs, programme implementation tools, health advice and guidelines. Cognizant of the data gap, they also developed a technical tool to set and monitor targets for key populations, which calls for disaggregation of health programming and data across five key population groups including transgender people.^7^^,^^8^ Drawing from a review of public health evidence and extensive research into human rights law at international, regional and national levels, the 2015 WHO report *Sexual health, human rights and law* explores ways to improve access to services. The report includes information on gender transition, how to provide gender-sensitive health-care services and how to reduce violence related to gender expression and identities.^9^

While transgender health is recognized as beyond sexual health, efforts to end the HIV epidemic – which have been driven by an explicitly rights-based approach and massive community mobilization – highlight the specific needs of transgender people These efforts enable policy-makers and advocates to navigate a contentious issue and to gather data and evidence that demonstrate the importance of more people-centred, tailored and informed responses.

The third shift is WHO’s adoption of an equity-focused and rights-based approach to health, including transgender health. Human rights standards call for the availability and accessibility of quality health information, including for transgender and other gender minorities, and require that all those seeking services should be treated with respect and dignity, free from discrimination. Rights-based approaches also require policy-makers to address the underlying determinants of health. The 2005–2008 Commission on Social Determinants of Health did not explicitly recognize gender identity in its final report.^10^ However, the WHO working group for monitoring action on the social determinants of health is considering the use of indicators that will monitor whether countries have implemented interventions that ensure the social inclusion of transgender people.^11^

WHO is also a party to United Nations (UN)-wide efforts to end discrimination, including against people with minority gender identities and sexual orientations. In 2015, WHO signed a joint statement with 11 other UN bodies calling for an end to discrimination and violence against gender and sexual minorities, including in health-care-settings.^12^ In 2016, The Joint United Nations Programme on HIV/AIDS and the WHO Global Health Workforce Alliance jointly launched the *Agenda for Zero Discrimination in Health Care*. The priorities of the agenda are to increase political commitment, to promote monitoring and evaluation of frameworks, build evidence, monitor progress, ensure accountability and foster the scale-up of effective actions. The agenda also calls for addressing the needs of health workers to provide decent and respectful care that reflects the values and preferences of the people they serve, including transgender people.^13^

The efforts described above provide some important lessons for how to tackle the challenges to ensuring transgender health and suggest some opportunities for further action. First, transgender people and communities must always be involved in health decision-making that affects them. Transgender people’s involvement and participation in mechanisms such as the WHO civil society reference group for HIV, surveys on values and preferences and consultations on the ICD, have made it possible to progress in addressing their health and well-being. Second, a coordinated approach to transgender health is critical to addressing transgender people’s specific health care and broader needs. The 2030 Agenda for Sustainable Development and its commitment to leaving no one behind, linked with WHO’s equity- and rights-based approach requires that health be addressed comprehensively and universally. These include the right to be free from discrimination, torture and inhuman and degrading treatment, autonomy and self-determination in accessing services and legal gender recognition. Only by recognizing and using a more holistic approach to health and its underlying determinants, will we achieve the sustainable development goals (SDGs).

Here we have described the efforts across WHO to address the health of transgender people. While these efforts are noteworthy, there is scope to do more. Given that this issue cuts across several aspects of health, equity, social determinants and rights, WHO should continue to work collaboratively across the relevant technical areas at global, regional and country level and together in collaboration with transgender communities. Addressing the health inequities defined by gender identity might well be a test of our ability to leave no one behind while achieving the SDGs. The efforts made so far are an important start.
